# Artificial Intelligent Multi-Modal Point-of-Care System for Predicting Response of Transarterial Chemoembolization in Hepatocellular Carcinoma

**DOI:** 10.3389/fbioe.2021.761548

**Published:** 2021-11-15

**Authors:** Zhongqi Sun, Zhongxing Shi, Yanjie Xin, Sheng Zhao, Hao Jiang, Dandan Wang, Linhan Zhang, Ziao Wang, Yanmei Dai, Huijie Jiang

**Affiliations:** Department of Radiology, The Second Affiliated Hospital of Harbin Medical University, Harbin, China

**Keywords:** hepatocellular carcinoma, artificial intelligence, computed tomography imaging, inflammation-based index, point-of-care predicting

## Abstract

Hepatocellular carcinoma (HCC) ranks the second most lethal tumor globally and is the fourth leading cause of cancer-related death worldwide. Unfortunately, HCC is commonly at intermediate tumor stage or advanced tumor stage, in which only some palliative treatment can be used to offer a limited overall survival. Due to the high heterogeneity of the genetic, molecular, and histological levels, HCC makes the prediction of preoperative transarterial chemoembolization (TACE) efficacy and the development of personalized regimens challenging. In this study, a new multi-modal point-of-care system is employed to predict the response of TACE in HCC by a concept of integrating multi-modal large-scale data of clinical index and computed tomography (CT) images. This multi-modal point-of-care predicting system opens new possibilities for predicting the response of TACE treatment and can help clinicians select the optimal patients with HCC who can benefit from the interventional therapy.

## Introduction

Liver cancer is the second most lethal tumor after pancreatic cancer and ranks the fourth leading cause of cancer-related death worldwide (Craig et al., 2020; Villanueva et al., 2019; Tao et al., 2020). In China, the 5-year survival rates have been reported to be 12% (Zheng et al., 2018). Hepatocellular carcinoma (HCC), which is the most common form of liver cancer (∼90% of liver cancer), remains a health challenge in the world (Llovet et al., 2021; Yu et al., 2020). In order to predict the prognosis of patients with HCC, the Barcelona Clinic Liver Cancer (BCLC) staging classification, which is approved by European Association for the Study of the Liver (EASL) and American Association for the Study of Liver Diseases (AASLD), has emerged as the standard classification in recent years (Llovet et al., 2008; Vitale et al., 2011; Yang et al., 2012). However, HCC is commonly at intermediate tumor stage (BCLC stage B) or advanced tumor stage (BCLC stage C), in which only some palliative treatment can be used to offer a limited overall survival (∼11–20 months) (Llovet et al., 2002; Hucke et al., 2011; Sieghart et al., 2015). According to international guidelines, transarterial chemoembolization (TACE) is the recommended treatment for Barcelona stage B patients with localized liver disease and good liver function (Camma et al., 2002; Otto et al., 2006; Takayasu et al., 2006). However, HCC is highly heterogeneous at the genetic, molecular, and histological levels, which makes the prediction of preoperative TACE efficacy and the development of personalized regimens challenging. Therefore, there are growing demands for exploiting a method to accurately predict response of TACE in HCC. Imaging setting, which included ultrasound, computed tomography (CT), and magnetic resonance imaging (MRI), can be a promising tool for the detection stage and risk assessment of HCC (Banerjee et al. (2015); Woodall et al., 2007). Due to the high sensitivity, worldwide availability, and easy interpretability, CT is still the most commonly used in the field of response of TACE therapy. The best response of TACE cannot always be achieved after one session of CT imaging, especially for patients with large tumors. However, multiple CT examinations can easily damage the liver function of patients. Therefore, other clinical evaluation indexes should be added to build a point-of-care predicting system for improving the predicting accuracy of TACE responses. Crucially, inflammation has been recognized as a major role in the tumorigenic process for HCC. Recent studies confirm that inflammation also plays a prognostic role in the whole clinical process of malignancy (Sanghera et al., 2019; Chan et al., 2020; Wang et al., 2021). A number of inflammation-based indexes (IBIs) are derived from peripheral blood counts for prognostic purposes, with examples including neutrophil-to-lymphocyte ratio (NLR), platelet-to-lymphocyte ratio (PLR), monocyte-to-lymphocyte ratio (MLR), systemic immune-inflammation index (SII), and neutrophil-to-lymphocyte ratio (SIRI) (Pinato et al., 2012; Yang et al., 2020). Therefore, combined CT images with inflammation-based indexes to predict postoperative treatment responses and accurately identify patients who responded after TACE is of important clinical guiding significance. Recently, artificial intelligence (AI), which is capable of maximizing the predictive accuracy from static or dynamic data sources using analytic or probabilistic models, has markedly extended the reach of human beings in biomedical tasks (Esteva et al., 2017; Li et al., 2017; Kermany et al., 2018; Li et al., 2018; Chang et al., 2019; Zhang et al., 2020). Deep learning is especially recognized as demonstrating good performance for assessing radiological and recognizing images. Because of the multifactorial and complex nature of HCC, the convolutional neural network of deep learning algorithms has shown great potential in fully mining image information. This approach does not need to manually screen image features, and it shows good training performance for high-dimensional data processing (Gulshan et al., 2016; Peng et al., 2019; Liu et al., 2020). The texture analysis based on contrast-enhanced pretherapeutic dynamic CT may act as imaging biomarkers to predict response for HCC. Higher gray-level co-occurrence matrix and smaller tumor size are significant signs. However, the highest AUC was only 0.72 (Park et al., 2017; Kermany et al., 2018). It is necessary to find a new method to increase predicting accuracy of TACE responses. Because of the multifactorial and complex nature of HCC, building a deep learning point-of-care predicting system to integrate multiple factors (e.g., CT images and inflammation-based indexes) would appear to be a highly effective technique to autonomously predict the response of TACE therapy. In this paper, we aim to develop a point-of-care system for predicting the response of TACE in HCC by a concept of multi-modal large-scale data by combining clinical indexes with CT images. This multi-modal point-of-care predicting system opens new possibilities for predicting the response of TACE treatment and can help clinicians to select optimum patients with HCC who can benefit from the interventional therapy.

## Materials and Methods

### Patients

This study included patients in the Second Affiliated Hospital of Harbin Medical University. A total of 1,890 patients who underwent TACE were recruited from January 2011 to September 2020. Finally, 399 patients were enrolled. The patients who matched inclusion criteria were as follows: (1) Patients were diagnosed as HCCs *via* biopsy or radiological for the Study. (2) All patients did not have a history of previous TACE of HCC before CT examination. (3) Those who had hepatic-arterial CT imaging within 7 days before and 1 month after treatment. (4) Patients with BCLC stage B. The exclusion criteria were as follows: (1) Those with a history of previous TACE, liver transplantation, targeting therapy, radiotherapy, and palliative care treatment. (2) Patients with major thrombosis in portal vein or abdominal lymph node or distant metastases. (3) Other liver tumors that were confirmed with pathology or imaging. The response of hepatic-arterial CT images was classified into objective response [containing complete response (CR) and partial response (PR)] and non-response [containing progressive disease (PD) and stable disease (SD)] according to the modified Response Evaluation Criteria in Solid Tumors (mRECIST).

### CT Scan Protocols and Region-of-Interest Segmentation

Contrast-enhanced computed tomography (CECT) was performed with a 64-detector row scanner CT machine (GE Healthcare, United States). The scanning parameters were as follows: tube current, 250 mA; tube voltage, 120 kV; and slice thickness, 5 mm. Contrast agent (Ultravist, Bayer, Germany) for CECT was injected through a pump injector at a rate of 3.0 ml/s from the antecubital vein. Hepatic-arterial phase CT images were obtained at 35 s. All CT images were input into the Dr. wise AI software (Deepwise Inc., China). The regions of interest (ROIs) were delineated manually by two senior radiologists who had 15 years experience (reader 1, Prof. Huijie Jiang) and 13 years experience (reader 2, Prof. Jinling Zhang). The entire cohort included 399 patients who were randomly divided into a training dataset (319 cases) and validation dataset (80 cases) by a ratio of 8:2. The validation dataset evaluated the accuracy of the training dataset. The ROIs of CT images from the training cohort and the validation cohorts were manually segmented by the two readers who were specifically blinded to the therapy outcome of the patients.

### Image Analysis and Preprocessing

All CT original images were reconstructed using a post-processing workstation to achieve uniform slice thickness and input the reconstructed image into Deepwise software to delineate the ROIS. We saved one CT image and the corresponding mask of ROIs for each patient from the largest tumor area in hepatic-arterial phase CT images. ROI was delineated around the largest tumor area selected by transverse and sagittal observations, and the ROI area was outlined close to the edge of the tumor. A total of 319 patients were used as the training set and 80 patients were used as the validation set. Random image cropping and patching (RICAP) were employed for data augmentation for deep convolutional neural network training (Takahashi et al., 2018). The details are as follows: RICAP cropped new training CT images randomly from the original CT images and patched them to compose new training CT images set. Using this method, 5,460 patches were used to construct the new training set. In order to enhance the generalization ability of the model, the RICAP-based data augmentation was used in real time.

### Deep Learning Convolutional Neural Network

**FIGURE 1:**
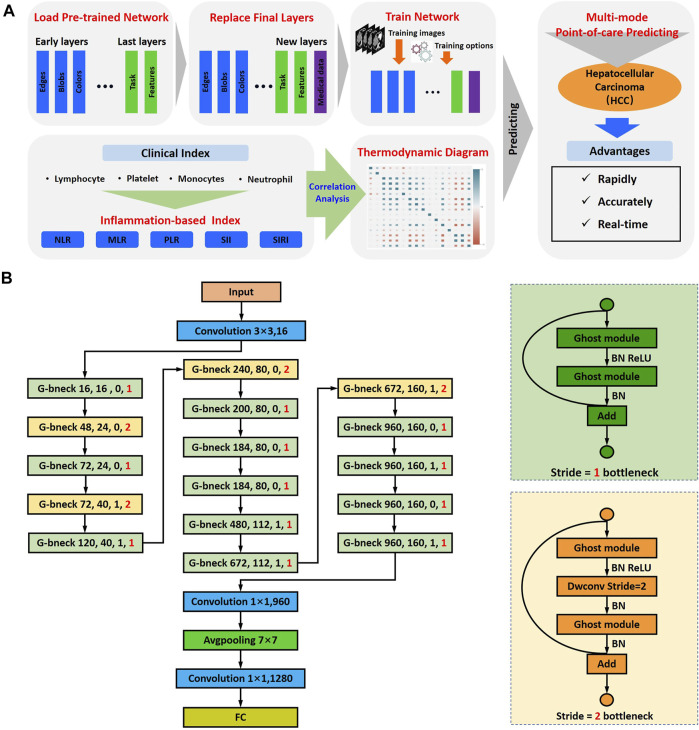
Schematic of multi-modal point-of-care predicting system. **(A)** Schematic of point-of-care system to predicting the response of TACE in HCC by integrating multi-modal large-scale CT imaging and clinical evaluation indexes. **(B)** The architecture of GhostNet and deep learning model flowchart.

GhostNet is an improved deep convolution neural network developed by Huawei Noah Ark Laboratory (Han et al., 2020; Paoletti et al., 2021). A GhostNet is a type of convolutional neural network that is built using Ghost modules, which aim to generate more features by using fewer parameters (allowing for greater efficiency). The architecture of GhostNet and the flowchart of deep learning for CT images are shown in Figure 1B. There are two important constituent concepts in the GhostNet. One is the Ghost module that can generate more feature maps from cheap operations. Through a series of linear transformations, ghost module can generate many ghost feature maps that can fully reveal the information behind the intrinsic features at a low computational cost. Another important concept is the Ghost bottleneck, which is designed to stack Ghost modules. The Ghost bottleneck appears to be the basic block in Ghostnet in which several convolutional layers and shortcuts are integrated. In general, the ghost bottleneck consists of two stacked Ghost modules. The first Ghost module expands the number of channels and the second Ghost module reduces the number of channels to match the shortcut path. Then, there is shortcut connected between the inputs and the outputs of these two Ghost modules. After each layer, the ReLU nonlinearity and batch normalization (BN) are applied, except that ReLU is not used after the second Ghost module. GhostNet mainly consists of a stack of Ghost bottlenecks that consist of the Ghost modules as the building block. Here, we clearly explain the meaning of the parameters of “G-bneck a, b, c, d” in the figure. The G-bneck denotes Ghost bottleneck, the first parameter “a” means expansion size, “b” means the number of output channels, “c” denotes whether using SE module, and “d” denotes the stride. The first layer is a standard convolutional layer with 16 filters and then a series of Ghost bottlenecks with gradually increased channels connected in turn. According to the sizes of their input feature maps, these Ghost bottlenecks are grouped into different stages, and all the Ghost bottlenecks in each stage are applied with stride = 1 except that the last one is with stride = 2. At the end of the Ghostnet, the global average pooling (7 × 7) and the convolutional layer are utilized to transform the feature maps to a 1,280-dimensional feature vector for final classification. In the Ghostnet, some ghost bottlenecks also contain the squeeze and excite (SE) module. However, there is no hard-swish nonlinearity function due to its large latency, which is different from the MobileNetV3.

### Implementation Details

Our implementation was based on the package for the Ghostnet Network in python (version 3.7.1). Our training experiments were performed in a Windows 10 environment on a computer server with the following specifications: CPU Intel Xeon Processor Platinum 8124 M at 3.00 GHz, GPU NVIDIA RTX 3060, and 128 GB RAM.

### Statistical Analysis

Statistical analyses were performed with R statistical software (R Core Team, 2018) and Origin 9.1 (OriginLab Corporation, United States). Categorical variables were described as frequency (percentage), use Ghostnet to perform 50 iterations on the data, and finally calculate the AUC value (95% confidence interval). The performance of the prediction model was evaluated with the area under the receiver operating characteristic (ROC) curve, and the confusion matrices were plotted in validation cohorts to calculate the accuracies of estimating the response of TACE therapy.

## Results

### Multi-Modal Point-of-Care Predicting System

In order to predict the response of TACE for HCC therapy, we developed a point-of-care system by a concept of integrating multi-modal large-scale data of CT imaging and clinical indexes (Figure 1A). This artificial intelligent predicting system could be divided into two parts: the computed tomography image-based predicting response of TACE and the clinical index-based evaluation of HCC therapy. The GhostNet, which was developed by Huawei Noah Ark Laboratory, was employed as a deep learning score for predicting the response of patients with HCC after treatment. The architecture of GhostNet and the flowchart of deep learning for CT images are shown in Figure 1B. The GhostNet consisted of two important constituent concepts. Firstly, the Ghost module could generate more feature maps from cheap operations. Secondly, the Ghost bottleneck was designed to stack Ghost modules. More details can be seen in *Materials and Methods*.

### Patients Clinical Characteristics

Our retrospective study had been approved by the institutional review board and Ethical Committee (KY 2019-217). Finally, 399 patients with HCC were enrolled in this study: 319 patients and 80 patients were allocated to the training cohort and validation cohort, respectively. Table 1 listed the detailed clinical characteristics of the two cohorts. In the training cohort, the age of 108 (33.7%) patients was more than 60 years, 87 (27.2%) were female patients, 289 (90.5%) of the patients were diagnosed with hepatitis B virus, patients with AST and ALT over 20 U/ml were 214 (67.1%) and 146 (45.8%), respectively. Patients with abnormal AFP were 167 (52.5%). Similarly, hepatitis B virus positive in the validation cohort was 77 (95.6%); patients with higher than normal AST, ALT, and AFP were 56 (69.7%), 28 (35.3%), and 40 (49.7%), respectively. As can be seen, no significant differences were observed between the training cohorts and validation cohorts in the clinical database.

**TABLE 1:** Participant characteristics in the training and validation cohorts.

Characteristic	Training cohort (*n* = 319)	Validation cohort (*n* = 80)
Age (years)		
≤60	211 (66.3%)	45 (56.5%)
>60	108 (33.7%)	35 (43.5%)
Sex		
Male	232 (72.8%)	55 (69.0%)
Female	87 (27.2%)	25 (31.0%)
HBsAg status		
Positive	289 (90.5%)	77 (95.6%)
Negative	30 (9.5%)	3 (4.4%)
Child–Pugh classification		
A	248 (77.9%)	64 (79.67%)
B	71 (22.1%)	16 (21.33%)
ALT (U/ml)		
≤40	173 (54.2%)	52 (64.7%)
>40	146 (45.8%)	28 (35.3%)
AST (U/ml)		
≤40	105 (32.9%)	24 (30.3%)
>40	214 (67.1%)	56 (69.7%)
AFP (ng/ml)		
≤20	152 (47.5%)	40 (50.3%)
>20	167 (52.5%)	40 (49.7%)
Hepatocirrhosis status		
Present	195 (61.0%)	44 (54.9%)
Absent	124 (39.0%)	36 (45.1%)
Response to therapy		
Objective response	184 (57.7%)	50 (62.5%)
Non-response	135 (42.3%)	30 (37.5%)

### Classification of the TACE Therapy Response

**FIGURE 2:**
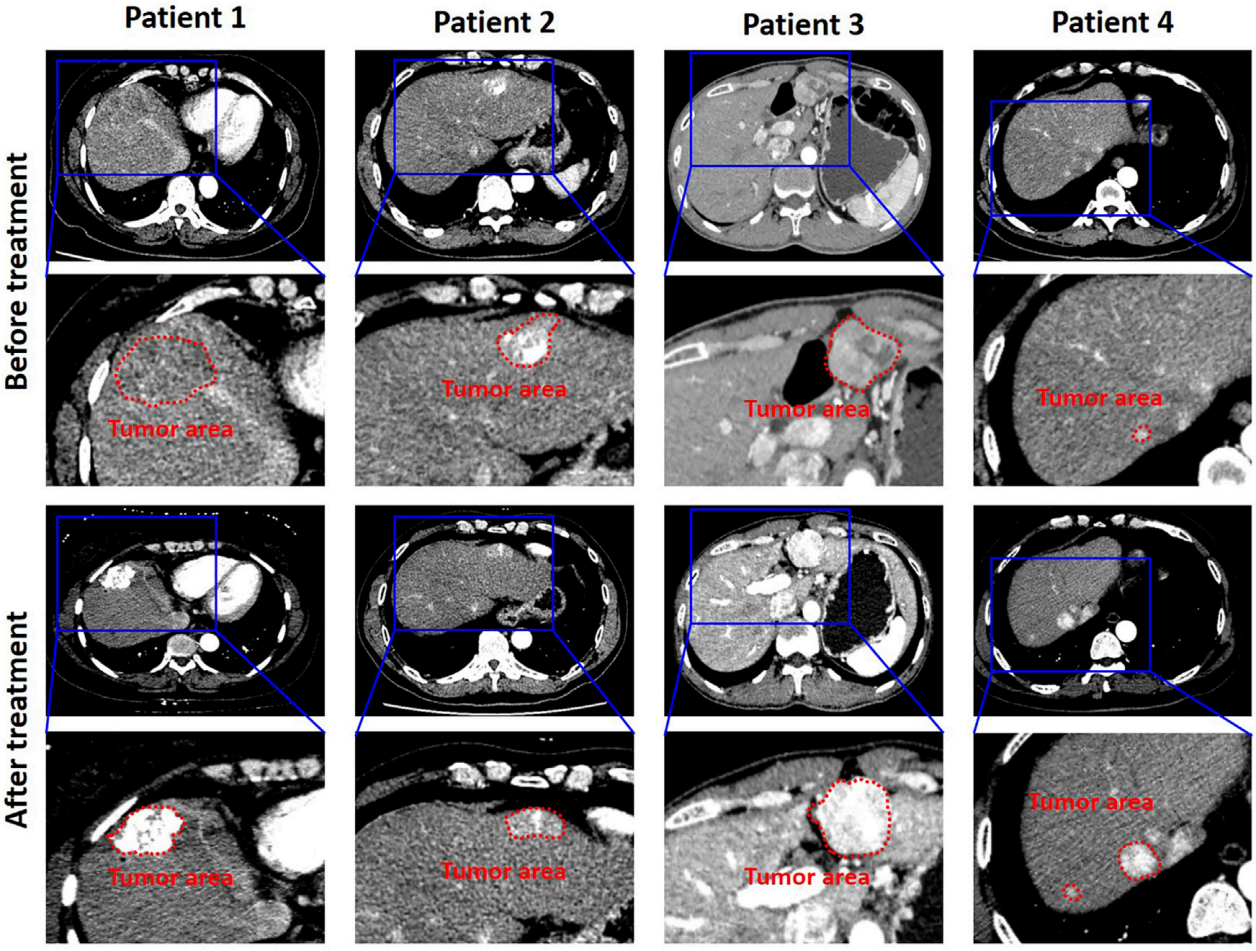
CT images were collected 7 days before treatment and 1 month after treatment, respectively. The measurement range was the longest diameter of the enhanced lesion in the tumor area. The tumor area of validation cohort with HCC of TACE therapy is shown in the red box.

Four typical CT images with different TACE responses from the validation cohort are shown in Figure 2. According to the mRECIST standards, the responses of patients after TACE treatment could be divided into two groups: the objective response and non-response. The objective response was defined as the tumor disappearance or the tumor area and corresponding cross-sectional diameter decreased at least 30%. After the TACE therapy, the cross-sectional diameter gradient of tumor of patients 1 and 2 were 43.9% (from 38.2 to 21.3 mm) and 40.7% (from 31.6 to 12.8 mm), respectively. Therefore, patients 1 and 2 were the typical objective response. The non-response was defined as the tumor area and corresponding cross-sectional diameter decreased less than 30% or the tumor progressed. Patients 3 and 4 belonged to the non-response. As can be seen, the cross-sectional diameter of tumor of patient 3 decreased from 32.5 to 30.7 mm (∼5.5%). Especially for patient 4, the cross-sectional diameter showed an increased trend (from 11.8 to 20.3 mm). In addition, a new tumor lesion occurred in the bottom of the left lobe of liver.

### Training and Validation of the Point-of-Care Predicting System

**FIGURE 3:**
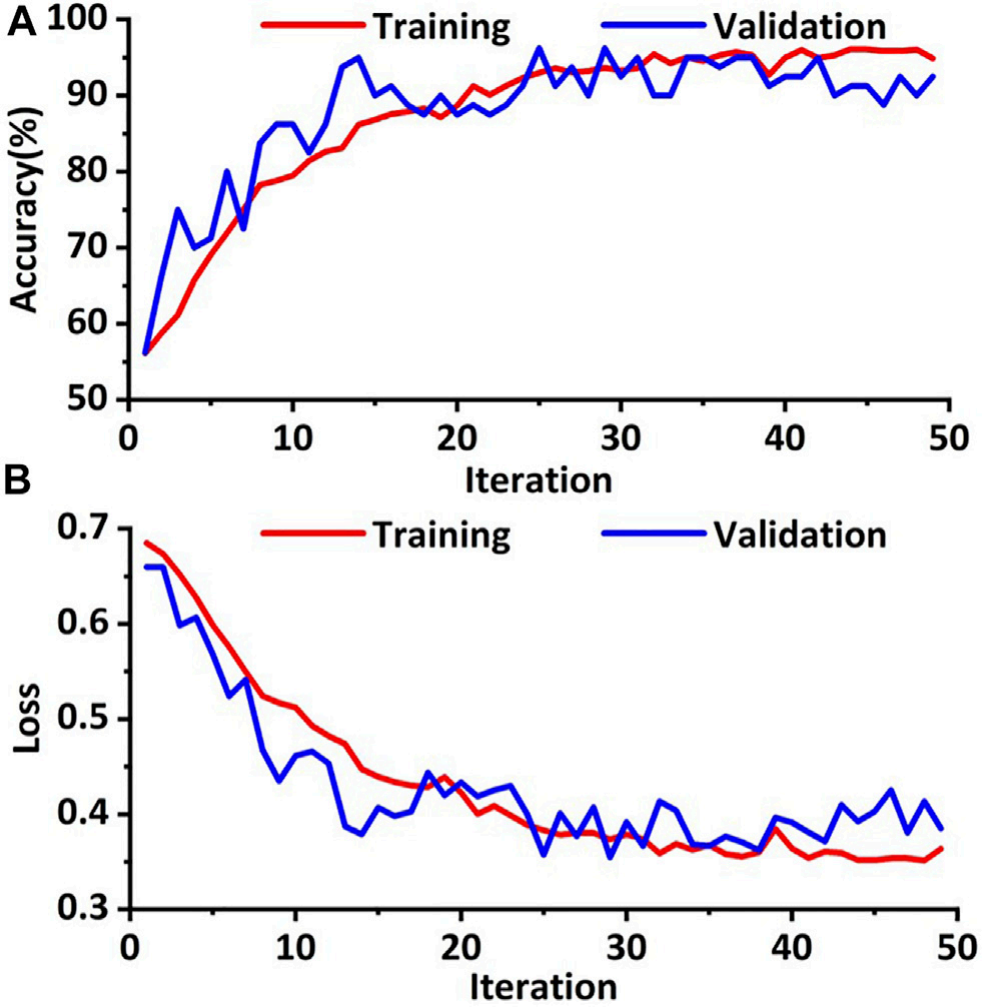
Training and validation processes of the deep learning model based on the CT images. **(A)** The accuracies of training and validation cohort *via* the ghost network model were approximately 98.0%. **(B)** The final training and validation losses were close to 0.4.

The training cohort was augmented through the way of RICP to avoid data overfitting. In order to increase the robustness of the model, an improved deep convolution neural network (GhostNet) was used for data training. As shown in Figures 3A,B, the accuracy of the improved deep learning model was approximately 98% and the cross-entropy loss was close to 0.4 after training (∼50 training epoch). These results indicated that the improved deep learning model showed good performance on distinguishing the response of TACE therapy in these cohorts.

**FIGURE 4:**
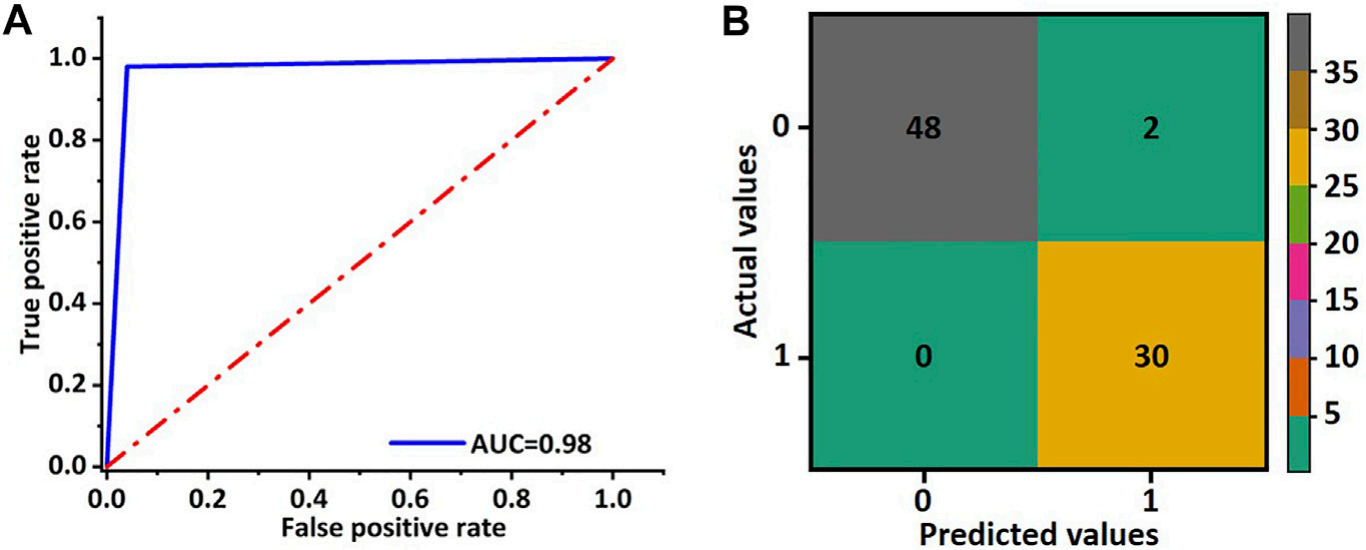
Receiver operating characteristic curve and confusion matrix of validation cohort for the ghost network. **(A)** The AUC value of the ROC was used to evaluate the training effect of the model. **(B)** The accuracy of the ghost model in image classification was evaluated by the obfuscation matrix.

In order to evaluate the training effect of the GhostNet based improved deep learning model, the AUC of the receiver operating characteristic curve was calculated. As shown in Figure 4A, the AUC of the deep learning model was about 0.98. The predictive accuracy of the deep learning model in each patch by confusion matrix after 50 epochs training was also investigated. The number of true-positive (TP) patches, false-positive (FP) patches false-negative (FN) patches, and true-negative (TN) patches were 30, 2, 0, and 48, respectively as shown in Figure 4B. Hence, the precision, F1 score, and accuracy were 0.94, 0.97, and 0.98, respectively. These results indicated that the improved deep learning model could increase the robust accuracy of predicting the TACE response.

### IBI-Based Predicting of TACE Response

**FIGURE 5:**
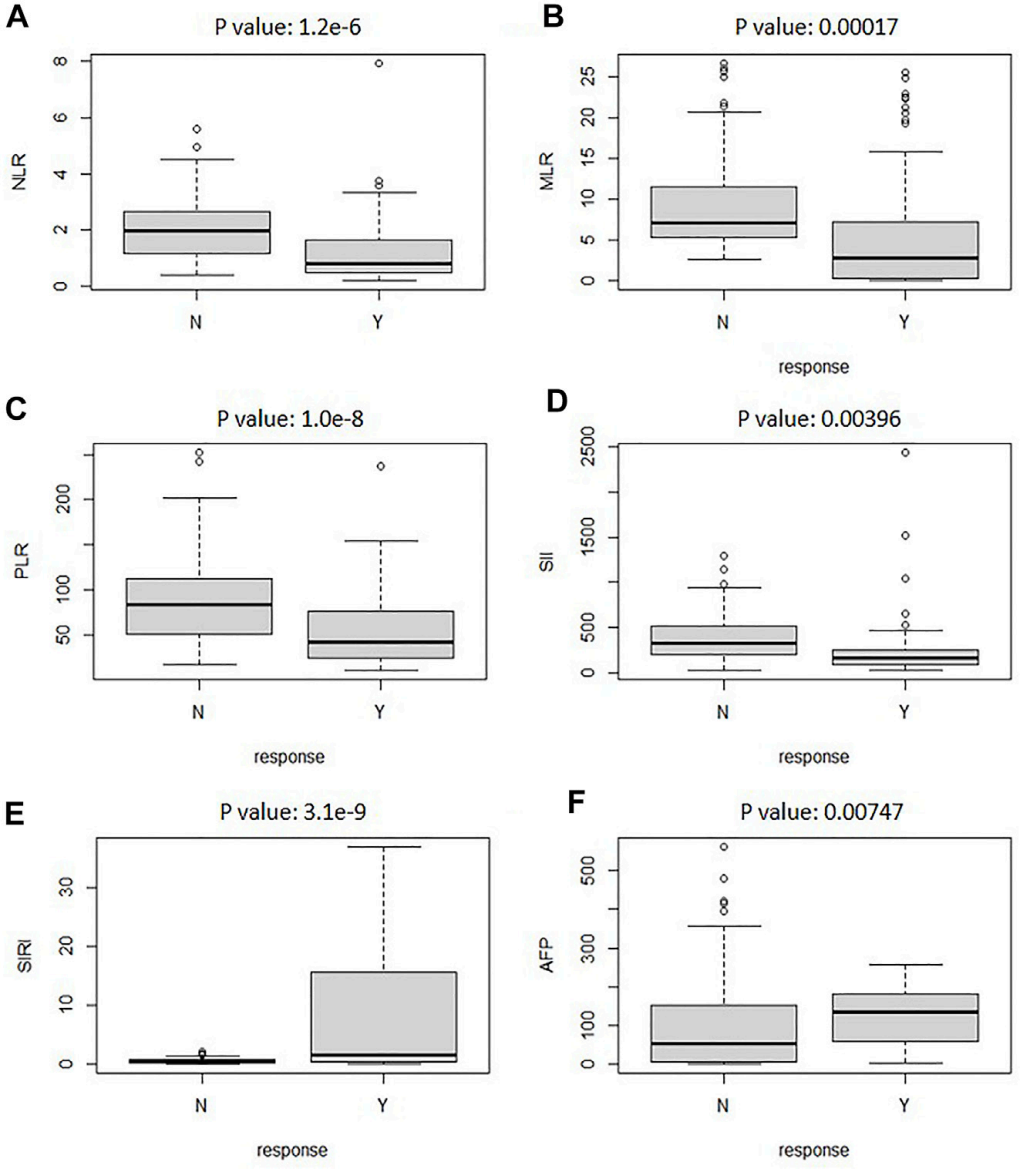
The boxplot analyzed the relationship between reactive and nonreactive values for clinical evaluation indexes of NLR **(A)**, AFP **(B)**, SIRI **(C)**, SII **(D)**, MLR **(E)**, and PLR **(F)**.

The best response of TACE could not always be achieved after one session of CT imaging, especially for patients with large tumors. In addition, the CT image could not be achieved frequently due to damage of ionizing radiation on the patients. Therefore, other evaluation clinical indexes should be added to this model for efficiently predicting the response of TACE therapy. Recent studies confirmed that IBI also plays a prognostic role in the whole clinical process of malignancies. Figure 5 illustrates the boxplot of the clinical evaluation indexes. As can be seen, the box and median of objective response for NLR was extremely larger than that of non-response. Hence, SIRI had significant association with the response relation of TACE. Other clinical evaluation indexes had a certain correlation with the response relation. All the *p* values of the factors are less than 0.05. It presented that there are significant differences between the evaluation values of the six factors in response and non-response.

**FIGURE 6:**
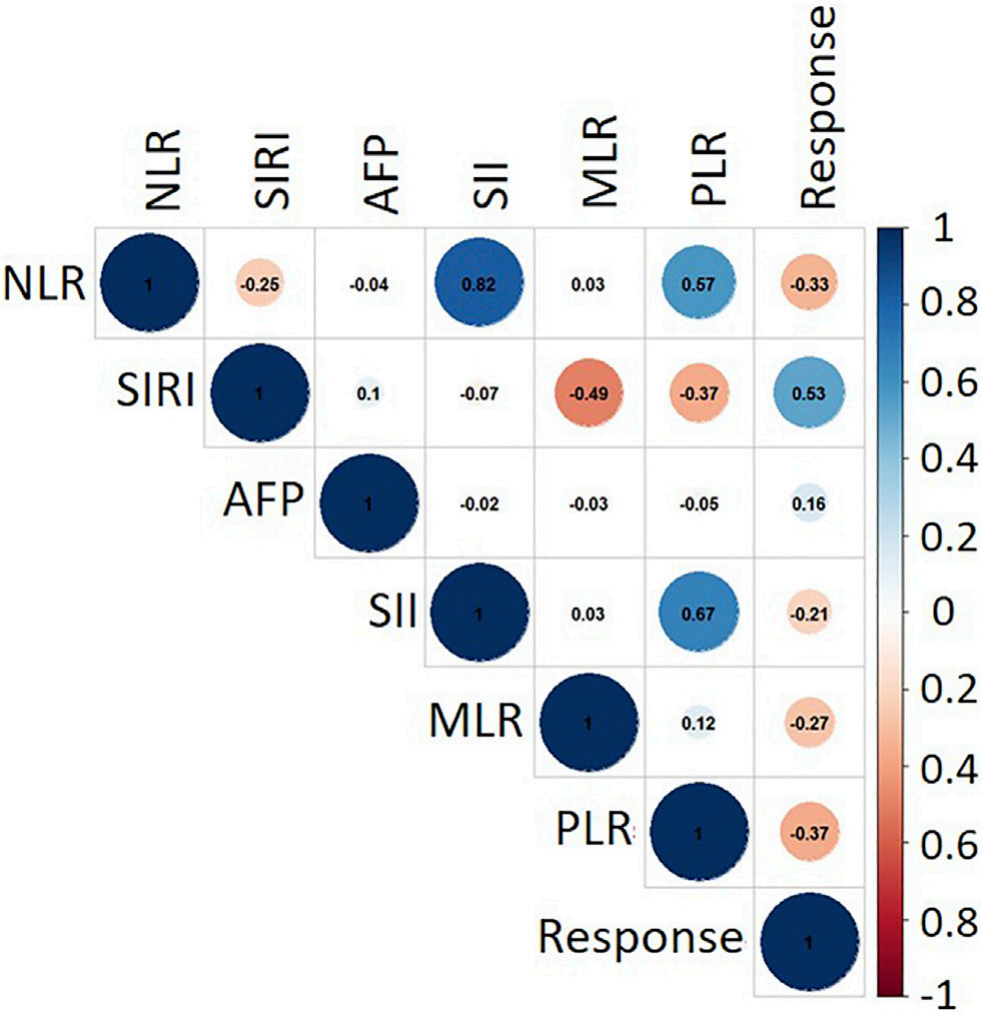
Correlation matrix between clinical evaluation indexes. The closer the absolute value of correlation coefficient was to 1, the stronger the correlation between variables was. The closer the absolute value of correlation coefficient was to 0, the weaker the correlation between variables was. The blue circle denotes a positive correlation, while red indicates a negative correlation.

To further investigate the correlation of clinical evaluation indexes with TACE therapy, the thermodynamic diagram was also achieved by statistical analysis. Figure 6 shows the association between the IBI and the objective response and non-response. Create a dummy variable for the reaction variable, and then the correlation was calculated; 1 represented the objective response. According to the correlation coefficient thermodynamic diagram, among the six factors, all factors had a certain correlation with the response relation, among which SIRI had a significant association with a correlation of 0.53, and AFP had no significant association with the response relation with a correlation of 0.16. Among them, PLR had a significant association. Hence, SIRI and PLR could be used to predict the response of TACE therapy.

## Discussion

In this study, we demonstrated a new artificial intelligent point-of-care multi-modal system for predicting the response of TACE therapy on patients with HCC. Based on integrating the multi-modal data of CT images and IBI, an improved deep learning model was employed to formulate precisely the interventional treatment plan for HCC (especially for patients at intermediate tumor stage or advanced tumor stage).

According to international guidelines, the TACE was recommended as the optimal treatment for patients with HCC at the intermediate tumor stage. The response of TACE therapy was crucial for clinicians to accurately identify patients who responded after TACE. Recent studies showed that CT imaging setting could be a promising tool for the detection, stage, and risk assessment of HCC (Woodall et al., 2007; Banerjee et al., 2015). In our study, 399 patients with HCC who underwent TACE had preoperative and postoperative CT enhanced images and clinical information, were enrolled to training a new deep learning model for predicting the response of TACE before operation. Using the random image cropping and patching method, 5,460 patches, which were cropped and patched from the original CT images, were used to construct a new training set. The accuracy of the improved deep learning model was approximately 98.0%, and the cross-entropy loss was close to 0.4. These results indicated that the improved deep learning model showed a good performance on the distinguishing of the response of TACE therapy in these cohorts.

However, CT was inapplicable for suspicious recurrence or atypical image. In addition, the CT images could not be achieved frequently due to damage of ionizing radiation on patients. Hence, it was necessary to find other clinical indexes combined with CT images for TACE response prediction. The included inflammation indexes (such as the NLR, MLR, PLR, SII, and SIRI) and AFP, which were derived from peripheral blood counts (e.g., neutrophil, lymphocyte, and platelet) and acute-phase proteins [e.g., C-reactive protein (CRP) and albumin], represented enabling tumor characteristics. Hence, task challenge remained about how to preferably integrate multi-modal data such as clinical index and CT images into an artificial intelligent system that enabled patients’ outlook to be predicted accurately. The studies showed that SIRI and PLR are significantly correlated with the response of TACE therapy.

We showed that this artificial intelligent point-of-care system integrating multi-modal data of CT images, SIRI, and PLR could be used for precisely predicting the response of the patients with HCC. However, our study has several limitations. First, this was a retrospective research. Second, multi-center prospective data will be collected for external verification of GhostNet performance in the following study. In the future, we would apply other feature selection techniques and clinical indexes (such as genes and proteins) to further improve the accuracy of diagnosis of HCC.

## Conclusion

In summary, a new artificial intelligent point-of-care multi-modal system based on CT images and clinical evaluation indexes would potentially serve as a new tool for predicting the response of TACE therapy on patients with HCC. The accuracy of this artificial intelligent system was approximately 98.0%, and the cross-entropy loss was close to 0.4. In addition, SIRI and PLR had a significant association with the responses of TACE therapy. These results indicated that this system showed a good performance on distinguishing the response of TACE therapy. This multi-modal point-of-care predicting system opened new possibilities to help clinicians select optimum patients with HCC who could benefit from the interventional therapy.

## Data Availability

The raw data supporting the conclusion of this article will be made available by the authors, without undue reservation.

## References

[B1] Banerjee S. Wang D. S. Kim H. J. Sirlin C. B. Chan M. G. Korn R. L. (2015). A Computed Tomography Radiogenomic Biomarker Predicts Microvascular Invasion and Clinical Outcomes in Hepatocellular Carcinoma. Hepatology 62, 792–800. 10.1002/hep.27877 25930992 PMC4654334

[B2] Cammà C. Schepis F. Orlando A. Albanese M. Shahied L. Trevisani F. (2002). Transarterial Chemoembolization for Unresectable Hepatocellular Carcinoma: Meta-Analysis of Randomized Controlled Trials. Radiology 224, 47–54. 10.1148/radiol.2241011262 12091661

[B3] Chan S. L. Wong L.-L. Chan K.-C. A. Chow C. Tong J. H.-M. Yip T. C.-F. (2020). Development of a Novel Inflammation-Based index for Hepatocellular Carcinoma. Liver Cancer 9, 167–181. 10.1159/000504252 32399431 PMC7206612

[B4] Chang X. Tang W. Feng Y. Yu H. Wu Z. Xu T. (2019). Coexisting Cooperative Cognitive Micro‐/Nanorobots. Chem. Asian J. 14, 2357–2368. 10.1002/asia.201900286 30989807

[B5] Craig A. J. von Felden J. Garcia-Lezana T. Sarcognato S. Villanueva A. (2020). Tumour Evolution in Hepatocellular Carcinoma. Nat. Rev. Gastroenterol. Hepatol. 17, 139–152. 10.1038/s41575-019-0229-4 31792430

[B6] Esteva A. Kuprel B. Novoa R. A. Ko J. Swetter S. M. Blau H. M. (2017). Dermatologist-level Classification of Skin Cancer with Deep Neural Networks. Nature 542, 115–118. 10.1038/nature21056 28117445 PMC8382232

[B7] Gulshan V. Peng L. Coram M. Stumpe M. C. Wu D. Narayanaswamy A. (2016). Development and Validation of a Deep Learning Algorithm for Detection of Diabetic Retinopathy in Retinal Fundus Photographs. JAMA 316, 2402–2410. 10.1001/jama.2016.17216 27898976

[B8] Han K. Wang Y. Tian Q. Guo J. Xu C. Xu C. (20202020). Ghostnet: More Features from Cheap Operations. Proc. IEEE/CVF Conf. Comput. Vis. Pattern Recognition (Cvpr), 1580–1589. 10.1109/cvpr42600.2020.00165

[B9] Hucke F. Sieghart W. Schöniger-Hekele M. Peck-Radosavljevic M. Müller C. (2011). Clinical Characteristics of Patients with Hepatocellular Carcinoma in Austria - Is There a Need for a Structured Screening Program? Wien. Klin. Wochenschr. 123, 542–551. 10.1007/s00508-011-0033-9 21800047

[B10] Kermany D. S. Goldbaum M. Cai W. Valentim C. C. S. Liang H. Baxter S. L. (2018). Identifying Medical Diagnoses and Treatable Diseases by Image-Based Deep Learning. Cell 172, 1122–1131. 10.1016/j.cell.2018.02.010 29474911

[B11] Li T. Chang X. Wu Z. Li J. Shao G. Deng X. (2017). Autonomous Collision-free Navigation of Microvehicles in Complex and Dynamically Changing Environments. ACS Nano 11, 9268–9275. 10.1021/acsnano.7b04525 28803481

[B12] Li T. Zhang A. Shao G. Wei M. Guo B. Zhang G. (2018). Janus Microdimer Surface Walkers Propelled by Oscillating Magnetic fields. Adv. Funct. Mater. 28, 1706066. 10.1002/adfm.201706066

[B13] Liu Q. P. Xu X. Zhu F. P. Zhang Y. D. Liu X. S. (2020). Prediction of Prognostic Risk Factors in Hepatocellular Carcinoma with Transarterial Chemoembolization Using Multi-Modal Multi-Task Deep Learning. EClinicalMedicine 23, 100379. 10.1016/j.eclinm.2020.100379 32548574 PMC7284069

[B14] Llovet J. M. Di Bisceglie A. M. Bruix J. Kramer B. S. Lencioni R. Zhu A. X. (2008). Design and Endpoints of Clinical Trials in Hepatocellular Carcinoma. J. Natl. Cancer Inst. 100, 698–711. 10.1093/jnci/djn134 18477802

[B15] Llovet J. M. Kelley R. K. Villanueva A. Singal A. G. Pikarsky E. Roayaie S. (2021). Hepatocellular Carcinoma. Nat. Rev. Dis. Primers. 7, 6. 10.1038/s41572-020-00240-3 33479224 PMC13537433

[B16] Llovet J. M. Real M. I. Montaña X. Planas R. Coll S. Aponte J. (2002). Arterial Embolisation or Chemoembolisation versus Symptomatic Treatment in Patients with Unresectable Hepatocellular Carcinoma: a Randomised Controlled Trial. The Lancet 359, 1734–1739. 10.1016/s0140-6736(02)08649-x 12049862

[B17] Otto G. Herber S. Heise M. Lohse A. W. Mönch C. Bittinger F. (2006). Response to Transarterial Chemoembolization as a Biological Selection Criterion for Liver Transplantation in Hepatocellular Carcinoma. Liver Transpl. 12, 1260–1267. 10.1002/lt.20837 16826556

[B18] Paoletti M. E. Haut J. M. Pereira N. S. Plaza J. Plaza A. (2021). Ghostnet for Hyperspectral Image Classification. IEEE Trans. Geosci. Remote Sensing, 1–16. 10.1109/TGRS.2021.3050257

[B19] Park H. J. Kim J. H. Choi S.-y. Lee E. S. Park S. J. Byun J. Y. (2017). Prediction of Therapeutic Response of Hepatocellular Carcinoma to Transcatheter Arterial Chemoembolization Based on Pretherapeutic Dynamic CT and Textural Findings. Am. J. Roentgenology 209, W211–W220. 10.2214/ajr.16.17398 28813195

[B20] Peng J. Kang S. Ning Z. Deng H. Shen J. Xu Y. (2019). Residual Convolutional Neural Network for Predicting Response of Transarterial Chemoembolization in Hepatocellular Carcinoma from CT Imaging. Eur. Radiol. 30, 413–424. 10.1007/s00330-019-06318-1 31332558 PMC6890698

[B21] Pinato D. J. Stebbing J. Ishizuka M. Khan S. A. Wasan H. S. North B. V. (2012). A Novel and Validated Prognostic index in Hepatocellular Carcinoma: The Inflammation Based index (IBI). J. Hepatol. 57, 1013–1020. 10.1016/j.jhep.2012.06.022 22732513

[B22] Sanghera C. Teh J. J. Pinato D. J. (2019). The Systemic Inflammatory Response as a Source of Biomarkers and Therapeutic Targets in Hepatocellular Carcinoma. Liver Int. 39, 2008–2023. 10.1111/liv.14220 31433891

[B23] Sieghart W. Hucke F. Peck-Radosavljevic M. (2015). Transarterial Chemoembolization: Modalities, Indication, and Patient Selection. J. Hepatol. 62, 1187–1195. 10.1016/j.jhep.2015.02.010 25681552

[B24] Takahashi R. Matsubara T. Uehara K. (2018). Data Augmentation Using Random Image Cropping and Patching for Deep CNNs. IEEE Trans. Circuits Syst. Video Technol. 30, 2917–2931.

[B25] Takayasu K. Arii S. Ikai I. Omata M. Okita K. Ichida T. (2006). Prospective Cohort Study of Transarterial Chemoembolization for Unresectable Hepatocellular Carcinoma in 8510 Patients. Gastroenterology 131, 461–469. 10.1053/j.gastro.2006.05.021 16890600

[B26] Tao Y. Wang J. Xu X. (2020). Emerging and Innovative Theranostic Approaches for Mesoporous Silica Nanoparticles in Hepatocellular Carcinoma: Current Status and Advances. Front. Bioeng. Biotechnol. 8, 184. 10.3389/fbioe.2020.00184 32211399 PMC7075945

[B27] Villanueva A. (2019). Hepatocellular Carcinoma. N. Engl. J. Med. 380, 1450–1462. 10.1056/nejmra1713263 30970190

[B28] Vitale A. Ramirez Morales R. Zanus G. Farinati F. Burra P. Angeli P. (2011). Barcelona Clinic Liver Cancer Staging and Transplant Survival Benefit for Patients with Hepatocellular Carcinoma: a Multicentre, Cohort Study. Lancet Oncol. 12, 654–662. 10.1016/s1470-2045(11)70144-9 21684210

[B29] Wang Z.-C. Jiang W. Chen X. Yang L. Wang H. Liu Y.-H. (2021). Systemic Immune-Inflammation index Independently Predicts Poor Survival of Older Adults with Hip Fracture: a Prospective Cohort Study. BMC. Geriatr. 21, 155. 10.1186/s12877-021-02102-3 33663402 PMC7934427

[B30] Woodall C. E. Scoggins C. R. Loehle J. Ravindra K. V. McMasters K. M. Martin R. C. G. (2007). Hepatic Imaging Characteristics Predict Overall Survival in Hepatocellular Carcinoma. Ann. Surg. Oncol. 14, 2824–2830. 10.1245/s10434-007-9525-2 17690939

[B31] Yang J. Bao Y. Chen W. Duan Y. Sun D. (2020). Nomogram Based on Systemic Immune Inflammation Index and Prognostic Nutrition Index Predicts Recurrence of Hepatocellular Carcinoma after Surgery. Front. Oncol. 10, 551668. 10.3389/fonc.2020.551668 33163397 PMC7591400

[B32] Yang T. Lin C. Zhai J. Shi S. Zhu M. Zhu N. (2012). Surgical Resection for Advanced Hepatocellular Carcinoma According to Barcelona Clinic Liver Cancer (BCLC) Staging. J. Cancer Res. Clin. Oncol. 138, 1121–1129. 10.1007/s00432-012-1188-0 22402598 PMC11824283

[B33] Yu L. Xu F. Gao L. (2020). Predict New Therapeutic Drugs for Hepatocellular Carcinoma Based on Gene Mutation and Expression. Front. Bioeng. Biotechnol. 8, 8. 10.3389/fbioe.2020.00008 32047745 PMC6997129

[B34] Zhang Z.-M. Tan J.-X. Wang F. Dao F.-Y. Zhang Z.-Y. Lin H. (2020). Early Diagnosis of Hepatocellular Carcinoma Using Machine Learning Method. Front. Bioeng. Biotechnol. 8, 254. 10.3389/fbioe.2020.00254 32292778 PMC7122481

[B35] Zheng R. Qu C. Qu C. Zhang S. Zeng H. Sun K. (2018). Liver Cancer Incidence and Mortality in China: Temporal Trends and Projections to 2030. Chin. J. Cancer Res. 30, 571–579. 10.21147/j.issn.1000-9604.2018.06.01 30700925 PMC6328503

